# A national cross-sectional study for poliovirus seroprevalence in the Republic of Korea in 2012: implication for deficiency in immunity to polio among middle-aged people

**DOI:** 10.1186/s12879-015-0894-z

**Published:** 2015-03-28

**Authors:** Hye-Jin Kim, Seoyeon Hwang, Somin Lee, Yunhyung Kwon, Kwangsook Park, Young Joon Park, Geun-Ryang Bae, Sang Won Lee, Yong-Seok Jeong, Ji-Yeon Hyeon

**Affiliations:** WHO Polio National Reference Laboratory, Division of Vaccine Research, Center for Infectious Diseases, Korea National Institutes of Health, Korea Centers for Disease Control and Prevention, Osong-eup, Cheongju, Chungcheongbuk-do 363-951 Republic of Korea; Division of Vaccine Preventable Disease Control and Prevention, Center for Infectious Diseases Control, Korea Centers for Disease Control and Prevention, Osong-eup, Cheongju, Chungcheongbuk-do 363-951 Republic of Korea; Department of Biology Graduate School, Kyung Hee University, Seoul, Dongdaemon-gu 130-701 Republic of Korea; Current Address: Division of HIV/AIDS and TB Control, Center for Infectious Diseases Control, Korea Centers for Disease Control and Prevention, Osong-eup, Cheongju, Chungcheongbuk-do 363-951 Republic of Korea

**Keywords:** Poliovirus, Neutralization antibody, Seroprevalence, Inactivated polio vaccine

## Abstract

**Background:**

A worldwide poliomyelitis eradication program was initiated in 1988; however, strains of wild poliovirus (WPV) are still endemic in some countries. Until WPV transmission is eradicated globally, importation and outbreaks of WPV are alarming possibilities. This study is the first report to document the polio immunity after 2004, when an inactivated polio vaccine (IPV) was introduced in the Republic of Korea.

**Methods:**

A total of 745 serum samples from randomly selected patients ranging from 6 to 84 years of age were used for neutralization tests, performed in the World Health Organization polio national reference laboratory.

**Results:**

Among the 745 tested sera, 439 (58.9%) were seropositive and 19 (2.6%) were seronegative to all PV serotypes. In all age groups, PV3 showed the lowest level of seroprevalence, at 509 cases (68.3%), compared to 616 (82.7%) for PV1 and 685 (91.9%) for PV2. In the 6–10-year age group, which included IPV-immunized children, the highest seropositive rate was observed and the difference in seroprevalence between PV3 and other serotypes was the lowest compared to the other age groups immunized with oral PV vaccines (OPV). In addition, the seronegative rates of all three PV types in children aged 6–10 in this study were found to be lower than those in OPV-immunized children reported in a previous study from the Republic of Korea. Meanwhile, middle-aged subjects (41–60 years) had the lowest seroprevalence and geometric mean titer.

**Conclusions:**

This study indicates a deficiency in immunity to PV in middle-aged individuals, and low seroprevalence to PV3 in all age groups. In addition, due to the ongoing risk of importing PV, middle-aged people should consider PV vaccination before visiting a PV-endemic country. Our findings provide data to assist those involved in deciding future national polio vaccination strategies for the maintenance of a polio-free status in Korea.

## Background

Poliovirus (PV) is a member of the *Picornaviridae* family and is classified as a human enterovirus-C species of the genus *Enterovirus* [1,2]. PV can be divided into 3 different serotypes, PV1, PV2, and PV3, and can cause poliomyelitis and other neurologic disorders, as might be found with serious infectious diseases, and can cause permanent residual paralysis. PV is transmitted primarily through the fecal-oral route and can cause viremia following replication in the gastrointestinal tract [3,4]. Occasionally, PV invades the central nervous system and destroys lower motor neurons, causing a clinically distinctive flaccid paralysis [5].

Efforts to eradicate PV through a World Health Organization (WHO) vaccination program were initiated in 1988 [5], and eradication was achieved through intensive immunization and attentive surveillance [6]. However, wild PV (WPV) 1 and WPV3 are still currently endemic in 3 countries (Afghanistan, Nigeria, and Pakistan), with diminished chains of transmission, while WPV2 was eradicated in 1999 [7,8]. Therefore, despite the intensive efforts in eradicating PV, PV-free countries remain at risk of WPV importation. For example, in 2011, a strain of WPV1 that was genetically linked to a virus currently circulating in Pakistan was isolated in China, where the last indigenous poliomyelitis case had occurred in 1994 [9]. This transmission indicated that WPV remains a risk for all countries until it is completely eradicated globally. In addition, in May 2014, the WHO declared an emergency regarding the spread of WPV, based on recommendations on the international spread of PV; thus, travelers are now urged to undergo PV vaccination if they intend to travel to endemic areas [http://www.polioeradication.org/Portals/0/Document/Emergency/PolioPHEICguidance.pdf]. Until WPV is eradicated globally, its importation and outbreaks will continue in countries that share borders with WPV-endemic countries, and adequate vaccination strategies are therefore essential in establishing protection against PV.

The Western-pacific region, including the Republic of Korea, was declared WPV-free in 2000. The oral PV vaccine (OPV), which contains the Sabin PV1, PV2, and PV3 strains, was introduced to the Republic of Korea in the early 1970s and included in the national immunization program [10]; at the end of 2004, it was replaced with the inactivated PV vaccine (IPV), which includes formalin-inactivated the Mahoney strain of PV1, the MEF-1 strain of PV2, and the Saukett strain of PV3 [7,10,11]. The immunization of PV is given three times to all infants at 2, 4, and 6–18 months of age, followed by a booster shot before school entry at between 4 and 6 years of age [10]. The immunization level for PV has been sustained at an estimated 90–95% since 1980 [12]; however, the seroprevalence of anti-PV antibodies has not been investigated in the Republic of Korea since the introduction of the IPV. Therefore, this study was designed to estimate the level of immunity against PV1, PV2, and PV3 in broad age groups, ranging from 6 to 84 years of age, in the Republic of Korea.

## Methods

### Study design

This study evaluated the seroprevalence of PV1, PV2, and PV3 antibodies, and the vaccination coverage in 745 serum samples selected from participants in regions throughout the Republic of Korea. The serum samples were collected from the study subjects between April and November 2012. Individuals who randomly attended hospitals for a blood test due to reasons not related to PV immunization were enlisted in the study, although the immunization history of the individuals was not available.

Approximately 2 mL of blood was collected, allowed to clot, and centrifuged, and the serum was aspirated into a sterile cryovial. Subsequently, the serum samples were transported to the WHO polio national reference laboratory of the Korea Centers for Disease Control and Prevention for analysis. All sera were stored at -20°C until tested. Before testing, each serum sample was inactivated at 56°C for 30 min.

Participant information (sex, age, and region) was collected. On enrollment, the subjects (range: 6–84 years of age) were stratified into five age groups: 6–10, 11–20, 21–40, 41–60, and >60 years. Seroprevalence was compared between the different groups in terms of sex, age, and region.

### Neutralization tests

Neutralization tests were performed using Sabin PV1, PV2, and PV3 strains and a rhabdomyosarcoma (RD) cell line according to the WHO manual [13]. The PV reference strains used in this study were 01/528 (Sabin I), 01/530 (Sabin II), and 01/532 (Sabin III), which were obtained from the WHO. The titers of neutralizing antibodies against PV1, PV2, and PV3 were determined using the micro neutralization assay [13]. The sera were inactivated at 56°C for 30 min and subsequently diluted from 1:4 to 1:2,048 in two-fold serial dilutions (final volume, 50 μl), in duplicate, and each dilution was incubated for 1 h at 36°C to allow the antibodies to bind to PV (100 TCID_50_). Next, the RD cell suspensions (1 × 10^5^ cells) were added to the virus-serum mixtures in 96-well plates. Virus and cell controls were included for comparison. The plates were incubated at 36°C and examined daily for 5 days for proof of cytopathic effect. Based on the WHO recommendations, serum with a titer of more than 1:8 was considered positive [14]. According to the previously defined standard [15,16], low, medium, and high immunity to PV1, PV2, and PV3 were defined as titers between 1:8–1:32, 1:64–1:256, and >1:512, respectively. The seroprotection rates and geometric mean titers (GMTs) of antibodies were calculated for each serotype in each age group.

### Data analysis

The GMTs (95% confidence intervals) for PV1, PV2, and PV3 antibodies among the groups were calculated. Statistical analysis was performed using SPSS version 15.0 (SPSS, Inc., Chicago, IL), with P-values < 0.05 considered statistically significant. Statistical significance was calculated using Chi-square test and linear-by-linear association.

### Ethics approval

The institutional review board of Korea Centers for Disease Control and Prevention approved the use of the samples (number: 2012-08EXP-07-R), and all patients provided written informed consent before enrolling in this study.

## Results

### Study population

Of the 745 subjects, 349 (46.8%) were male and 396 (53.2%) were female, with a male-to-female ratio of 1:1.13. On enrollment, the subjects were stratified into 5 age groups: 6–10 (*n* = 236; 31.7%), 11–20 (*n* = 216; 29.0%), 21–40 (*n* = 135; 18.1%), 41–60 (*n* =113; 15.2%), and >60 years (*n* = 45; 6.0%).

The geographical distribution of the study population was further analyzed according to the regions of Korea: Gyeonggi-do (*n* = 237; 31.8%), Gyeongsang-do (*n* = 170; 22.8%), Seoul (*n* = 126; 16.9%), Chungcheong-do (v = 112; 15.0%), Jeolla-do (*n* = 68; 9.1%), Gangwon-do (*n* = 24; 3.2%), and Jeju-do (*n* = 8; 1.1%) (Table 1).

**Table 1:** **Poliovirus-antibody seropositive subjects according to sex, age, and region (** ***n*** **= 745)**

		**PV1**		**PV2**		**PV3**	
	**No. of tested samples (%)**	**No. of positive samples (%)**	**P value** ^*^	**No. of positive samples (%)**	**P value**	**No. of positive samples (%)**	**P value**
Total	745	616 (82.7)		685 (91.9)		509 (68.3)	
Sex							
Male	349 (46.8)	289 (82.8)	0.51	314 (90.0)	0.04	236 (67.6)	0.38
Female	396 (53.2)	327 (82.6)		371 (93.7)		273 (68.9)	
Age (years)							
6–10 (IPV)	236 (31.7)	220 (93.2)	<0.01	228 (96.6)	<0.05	207 (87.7)	<0.01
11–20 (OPV)	216 (29.0)	173 (80.1)		199 (92.1)		126 (58.3)	
21–40 (OPV)	135 (18.1)	106 (78.5)		122 (90.4)		72 (53.3)	
41–60 (OPV)	113 (15.2)	79 (69.9)		95 (84.1)		74 (65.5)	
>60 (OPV)	45 (6.0)	38 (84.4)		41 (91.1)		30 (66.7)	
Region							
Gyeonggi-do	237 (31.8)	202 (85.2)	0.065	226 (95.4)	0.018	172 (72.6)	0.033
Gyeongsang-do	170 (22.8)	135 (79.4)		154 (90.6)		108 (63.5)	
Seoul	126 (16.9)	103 (81.7)		114 (90.5)		80 (63.5)	
Chungcheong-do	112 (15.0)	95 (84.8)		101 (90.2)		85 (75.9)	
Jeolla-do	68 (9.1)	54 (79.4)		59 (86.8)		42 (61.8)	
Gangwon-do	24 (3.2)	19 (79.2)		23 (95.8)		17 (70.8)	
Jeju-do	8 (1.1)	8 (100.0)		8 (100.0)		5 (62.5)	

^*^ *P* < 0.05 indicates significant differences of seroprevalence among the compared groups.

IPV, inactivated poliovirus vaccine; OPV, oral poliovirus vaccine; PV, poliovirus.

### Antibody seroprevalence

**Figure 1:**
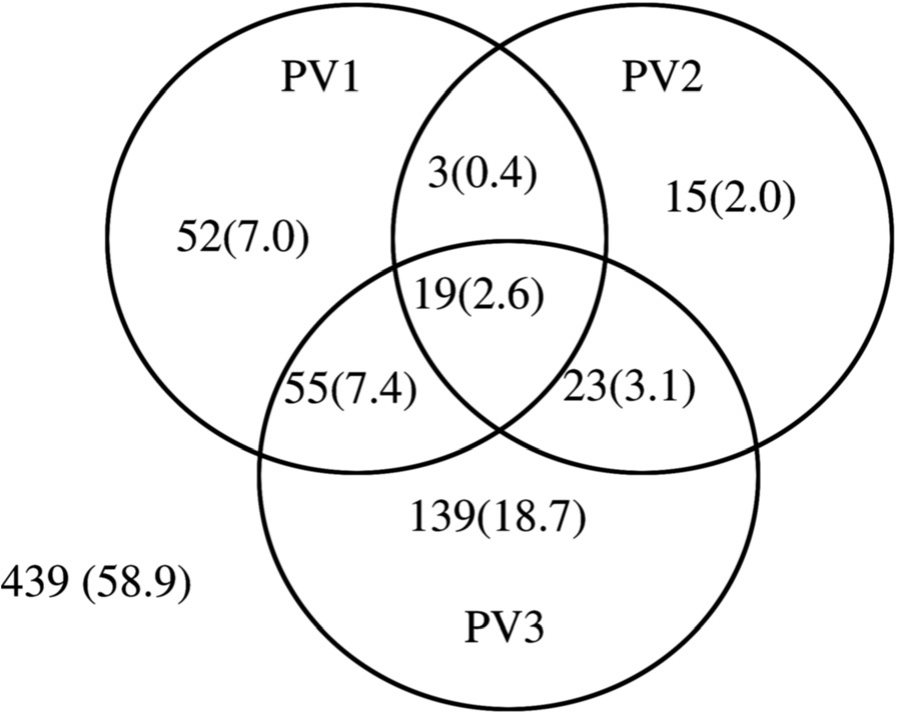
**The number (%) of subjects without neutralizing antibodies to one or more poliovirus (PV) types or a combination of PV1, PV2, and PV3.**

A total of 439 (58.9%) subjects were seropositive, and 19 subjects (2.6%) were seronegative to all three PV types (Figure 1). No detectable antibody was observed for 3 (0.4%), 7 (0.9%), 4 (0.5%), 5 (0.7%), and 0 patients in the 6–10, 11–20, 21–40, 41–60, and >60-year age groups, respectively (data not shown). The number of subjects seronegative to PV1, PV2, and PV3 were 52 (7.0%), 15 (2.0%), and 139 (18.7%), respectively. More subjects were seronegative to both the PV1 and PV3 antibodies (7.4%) than to both the PV2 and PV3 antibodies (3.1%) or both the PV1 and PV2 antibodies (0.4%) (Figure 1).

The numbers of seropositive subjects were as follows: 616 (82.7%) to PV1, 685 (91.9%) to PV2, and 509 (68.3%) to PV3. There was no significant difference in the number of subjects seropositive to PV1 and PV3 between the different sexes (P > 0.05); however, there was a significant sex difference for PV2 (P = 0.04) (Table 1).

In addition, there were significant correlations between the age groups and seropositivity. The 6–10-year age group showed the highest levels of seropositivity to PV1, PV2, and PV3 (93.2%, 96.6%, and 87.7%, respectively), while the 40–60-year age group had the lowest levels of seropositivity to PV1 and PV2 compared to the other age groups, including elderly people aged >60 years. For all age groups, the seropositivity was the lowest for PV3 and the highest for PV2. Furthermore, there was also significant regional differences in the seropositivity to PV2 and PV3 (P < 0.05). Jeolla-do had the lowest seroprevalence to both PV2 (86.8%) and PV3 (61.8%) compared with the other regions; meanwhile, Gyeonggi-do had the highest rates of seroprevalence for PV2 (95.4%) and PV3 (72.6%) (Table 1).

### Comparison of geometric mean titers

**Figure 2:**
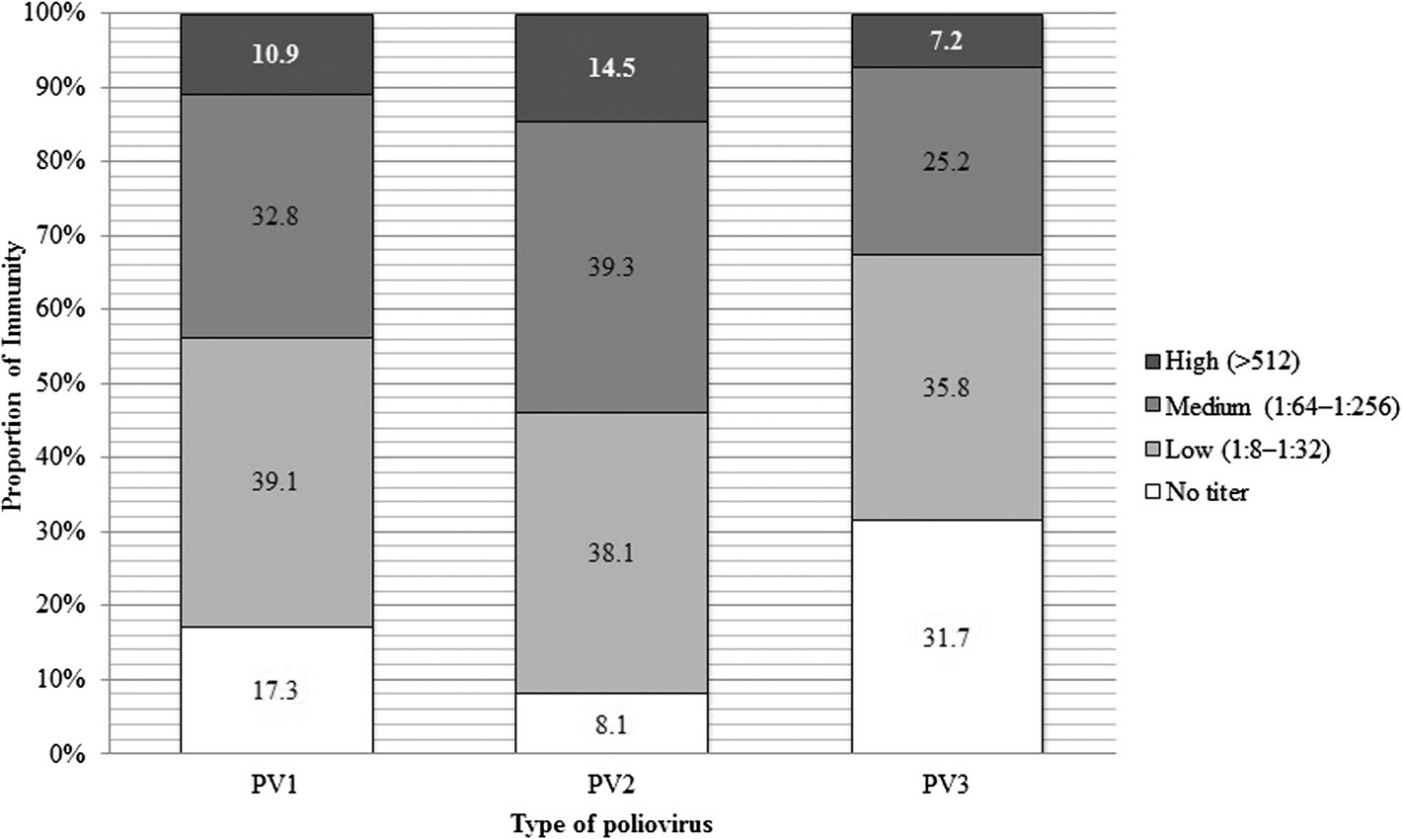
**Levels of immunity to poliovirus (PV) 1, PV2, and PV3.** Low, medium, and high immunity indicate neutralizing ranges of 1:8–1:32, 1:64–1:256, and >1:512. No immunity indicates that there was no titer.

The overall levels of immunity to PV are shown in Figure 2. The proportion of subjects with high immunity was 10.9% for PV1, 14.5% for PV2, and 7.2% for PV3. The corresponding proportions of medium immunity were 32.8%, 39.3%, and 25.2%, respectively, while those for low immunity were 39.1%, 38.1%, and 35.8%, respectively. Finally, the proportion of subjects with no immunity was the highest for PV3, at 31.7%, as compared to 17.3% for PV1 and 8.1% for PV2 (Figure 2).

The GMTs of PV are summarized in Table 2.

**Table 2:** **Geometric mean titers (GMTs) of poliovirus-antibodies according to sex, age, and region**

	**PV1**		**PV2**		**PV3**	
	**GMT**	**P value** ^*^	**GMT**	**P value**	**GMT**	**P value**
Total	163.1		206.2		108.4	
	(134.9–191.2)		(174.2–238.1)		(85.3–131.5)	
Sex						
Male	173.1	0.51	257.1	0.004	112.3	0.76
	(-37.6–75.4)		(32.3–159.5)		(-39.0–53.7)	
Female	154.2		161.2		105.0	
	(-37.4–75.3)		(30.5–161.4)		(-38.9–53.6)	
Age group (years)						
6–10	372.8	<0.01	470.2	<0.01	280.8	<0.01
	(295.9–449.7)		(383.0–557.4)		(213.5–348.1)	
11–20	83.0		107.1		38.2	
	(59.4–106.7)		(81.9–132.2)		(27.6–48.9)	
21–40	39.5		53.6		13.2	
	(27.6–51.3)		(39.9–67.2)		(9.5–16.9)	
41–60	34.0		63.5		23.9	
	(22.0–45.9)		(47.6–79.4)		(16.2–31.7)	
>60	142.6		113.2		38.9	
	(31.0–254.2)		(20.2–206.3)		(19.8–58.1)	
Region						
Gyeonggi-do	172.0	0.16	205.7	<0.01	100.3	<0.01
	(119.1–224.9)		(153.8–257.7)		(66.4–134. 3)	
Gyeongsang-do	176.0		248.2		128.1	
	(119.8–232.3)		(166.3–330.2)		(67.4–188.9)	
Seoul	109.6		100.1		40.0	
	(39.9–79.2)		(59.4–140.7)		(19.5–60.5)	
Chungcheong-do	218.0		273.6		185.5	
	(127.6–308.4)		(165.5–381.8)		(95.1–275.9)	
Jeolla-do	122.2		171.5		74.5	
	(70.3–174.1)		(92.2–250.8)		(33.9–115.0)	
Gangwon-do	149.3		263.7		135.0	
	(71.1–227.5)		(87.0–440.3)		(33.7–236.3)	
Jeju-do	86.0		173.0		136.0	
	(-58.2–230.2)		(-117.4–463.4)		(-164.1–436.1)	

The data are presented as mean (95% confidence interval).

^*^ *P* < 0.05 indicates significant differences of seroprevalence among the compared groups.

PV, poliovirus.

The GMTs of PV1, PV2, and PV3 were 163.1, 206.2, and 108.4, respectively. The lowest level of GMT was observed for PV3. Statistically significant differences in GMT were observed between the sexes for PV1 and PV3, and there were also significant differences in the GMTs between the age groups for each PV type (P < 0.01). The GMT for all PV types was the highest in the 6–10-years age group compared to the other groups. In particular, the GMTs of PV2 and PV3 in the 21–40-year age group were lower than those in the other groups. For PV1, the GMT was lower in the 41–60 years age group than that in the other groups. Higher GMTs were observed for PV2 than for the other PV types for all age groups, and lower GMTs were observed for PV3 for all age groups. For PV2 and PV3, there were statistically significant differences in the GMTs between the different regions, with the Chungcheong-do province having the highest overall GMTs for PV antibodies, while Seoul had the lowest GMT levels for PV2 and PV3 (Table 2).

## Discussion and conclusions

Although WPV has not been detected since Korea was declared WPV-free in 2001 [12], the Korean population could be at risk of infection through the importation of WPV or vaccine-derived PV, similar to what has been reported in England and Wales [17]. Therefore, in this study, we evaluated the seroprevalence of neutralizing antibodies to PV1, PV2, and PV3 in broad age groups in the Republic of Korea. To our knowledge, this is the first report to document the seroprevalence of antibodies against the three PV types since the introduction of IPV in the Republic of Korea. Since the introduction of the Sabin vaccine in 1962 and the inclusion of OPV vaccination in the National Immunization Program in 1970, the incidence of poliomyelitis has dramatically declined [10]. IPV is known to be safe and effective and to have played an important role in the control of poliomyelitis [18]. Accordingly, the Republic of Korea is committed to maintaining the present level of poliomyelitis immunization, especially since the introduction of IPV at the end of 2004.

We moreover compared the PV seroprevalence in the IPV-immunized children (6–10 years) in this study to that in OPV-immunized children (6–11 years) in a previous study from the Republic of Korea [10] (Table 3).

**Table 3:** **PV seroprevalence of IPV-immunized children compared to OPV-immunized children in a previous study** [10]

	**No. of samples (%)**				
	**All positive**	**PV1-negative**	**PV2-negative**	**PV3-negative**	**All negative**
IPV-immunized children (6–10 years, *n* = 236)	195 (82.6%)	16 (2.6%)	8 (1.0%)	29 (7.5%)	3 (1.3%)
OPV-immunized children [10] (6–11 years, *n* = 500)	411 (82.2%)	13 (6.8%)	5 (3.4%)	38 (12.3%)	0 (0%)
P value^*^	>0.05	<0.01	<0.05	<0.05	<0.05

^*^ *P* < 0.05 indicates significant differences of seroprevalence among the compared groups.

IPV, inactivated polio vaccine; OPV, oral poliovirus vaccine; PV, poliovirus.

The rates of full immunity to all PV types were similar, with 82.2% (411/500) of OPV-immunized children [10] and 82.6% (195/236) of IPV-immunized children (P > 0.05) showing full immunity. However, the seronegative rates of each PV type in IPV-immunized children were significantly lower than the corresponding rates in OPV-immunized children (P < 0.05), and the antibodies to PV3 were lower than those to the other two PV types in both studies [10] (Table 3). In addition, there were no detectable antibodies in 3 subjects (1.3%) in IPV-immunized children, as compared to in zero subjects in OPV-immunized children [10], and this difference was statistically significant (P <0.05). This difference could result from several mechanisms such as different immunogenicity of vaccine type, deficiency of host immunity in 1990s, or inappropriate storage or administration of OPV vaccine.

The highest seroprevalence and GMTs were observed in the 6–10-year age group in the present study. In addition, the difference in seroprevalence between PV3 and the other PV types was less here than for the other age groups. This is likely attributable to the PV vaccination program, in which PV vaccination in the Republic of Korea is initiated in 2-month-old babies, and boosted before school entry before 6 years of age. In addition, those in the 41–60-year age group had the lowest seroprotection levels and GMTs compared to the other age groups. This age group was born in 1950–1970, prior to the inclusion of PV vaccination in the National Immunization Program. Accordingly, those in the 41–60-year age group may lack serum antibodies. Decreasing immunity to PV in older age groups because of waning effect has also been reported in other studies [19], and elderly age groups would appear to be at risk of infection in the case of WPV importation [17]. However, this study indicated that the seroprotection level and GMT of subjects >60 years were higher than those of subjects in the 41–60-year age group, and we speculate that humoral neutralizing antibodies to PV in subjects aged >60 years may have been enhanced by a memory B cell immune response from a natural infection.

Differences in the population susceptibility or clinical symptoms of poliomyelitis between different age groups have not been described; however, several polio outbreaks affecting adults in WPV-free countries have been previously reported, while poliomyelitis is rarely observed in adults in such countries [20-22]. The older age groups may contribute to WPV transmission without clinical symptoms, and the WHO has therefore recommended older individuals to get vaccinated as part of the outbreak response [http://www.polioeradication.org/Portals/0/Document/Emergency/PolioPHEICguidance.pdf].

WPV2 has been eradicated worldwide, and has not been detected since the last case in India in 1999 [23]. On the other hand, PV1 and PV3 are currently endemic in several countries in Africa and Asia, with the most recent WPV3 isolated in November 2012 [http://www.polioeradication.org/Portals/0/Document/StrategicPlan/StratPlan2010_2012_ENG.pdf]. In this study, the immunity levels were the highest for PV2 and lowest for PV3 for all age groups. The high immunogenicity to PV2 could be attributed to the early PV2 eradication. Conversely, the deficiency in immunity to PV3 has previously been described in several studies [16,24-27]; it may be explained by a lower potency of PV3 antigens in the vaccine; thus, a booster dose may be required to improve PV3 immunity.

In conclusion, the results of the present study suggest that the PV3 component of the PV vaccine should be further evaluated. In addition, the danger of WPV infection has not been completely averted, and PV vaccination strategies are absolutely necessary to preserve our present PV-free status. In particular, due to the ongoing risk of importable PV, if middle-aged people are travelling to PV-endemic countries, they should be urged to consider PV vaccination. Our findings provide data to assist those involved in deciding future national PV vaccination strategies to maintain a poliovirus-free status.
